# When the home becomes a hazard: fall risks among older adults in Thailand’s coastal rural communities

**DOI:** 10.1186/s12889-025-26106-5

**Published:** 2026-01-10

**Authors:** Parnchon Chokprasit Jaiyuen, Pakavadee Rakthong, Thanwarat Chantanachai

**Affiliations:** 1 Department of Physical Therapy, School of Allied Health Sciences, Walailak University, Nakhon Si Thammarat, 80160 Thailand; 2 Movement Science and Exercise Research Center, Walailak University (MoveSE-WU), Nakhon Si Thammarat, 80160 Thailand; 3 Program in Environmental Health, Faculty of Science and Technology, Suratthani Rajabhat University, Surat Thani, 84100 Thailand; 4 Faculty of Physical Therapy, Mahidol University, Nakhon Pathom, 73170 Thailand

**Keywords:** Aged, Community-dwelling, Cross-sectional study, Environmental factors, Falls

## Abstract

**Objective:**

In Thailand, the incidence of falls is higher in rural areas compared to urban regions, primarily due to environmental factors. While studies have examined fall risk factors, few have investigated home environments in rural coastal areas and their impact on falls among older adults. This study sought to investigate the relationship between home environmental risk factors and falls among older adults living in coastal rural communities in Thailand, contributing to targeted fall prevention strategies.

**Methods:**

This cross-sectional study enrolled a total of 60 older adults, aged between 60 and 93 years, residing in rural coastal communities of Surat Thani Province. Participants were required to be Thai nationals living in their own homes, able to communicate independently with researchers, and willing to provide informed consent. Demographic and home environmental data were collected through structured interviews and corroborated by on-site observations of each participant’s home environment. A fall was operationally defined as having experienced at least one falling incident during the past six months. Descriptive statistics were applied to summarize participants’ characteristics, while Chi-square and Fisher’s exact tests were utilized to examine the association between socioeconomic and environmental factors and fall variables.

**Results:**

Within rural coastal communities, 17% (95% CI: 7.2%-26.1%) of older adults reported experiencing at least one fall during the preceding six months. Bivariate analyses were conducted to explore factors associated with fall occurrence. In these analyses, being unemployed was significantly associated with a higher prevalence of falls (*p* = 0.002), whereas having a mosquito net installed in the bedroom was associated with a reduced risk of falling (*p* = 0.031).

**Conclusion:**

Falls were relatively common among older adults living in rural coastal areas, and two such socioeconomic and environmental factors demonstrated significant associations with fall occurrence. These findings suggest that initiatives aimed at addressing unemployment-related vulnerabilities and promoting practical home environment modifications may be beneficial for reducing fall risk in these communities, although further research is required to confirm causal pathways and evaluate the effectiveness of such interventions.

## Introduction

 Globally, the rising older adult population has led to a substantial increase in fall-related incidents, imposing significant public health and economic challenges [1]. Likewise, in Thailand, the population of individuals aged 60 years and above rose from 7% in 1990 to approximately 20% in 2022, demonstrating a rapid demographic transition toward an aging society [2]. This shift toward an aged society has been accompanied by a rise in concerns related to falls. In Thailand, falls among older adults result in over 60,000 hospitalizations each year, with an average of four fall-related deaths occurring daily [3]. These incidents often lead to disability, leading to psychological distress, fear of falling, increased dependency, and a significant raises annual healthcare costs [2].

Fall risk factors among older adults are commonly attributed to intrinsic and extrinsic risk factors [4]. Intrinsic factors include age-related physiological changes, chronic health conditions, balance and gait impairments, and cognitive decline [5, 6]. Extrinsic factors, on the other hand, pertain to environmental hazards such as slippery floors, uneven terrain, poor lighting, and lack of assistive infrastructure [7]. While intrinsic factors have been widely studied, growing attention is being directed toward the role of the environment, particularly in the rural area [7]. 

Residing in rural areas constitutes a significant risk factor contributing to the occurrence of falls [8]. In Thailand, the risk of falls is particularly elevated in rural regions, where older individuals often reside in homes with structural hazards such as split-level flooring, poor lighting, and unstable staircases [9]. Recent study conducted in northeastern Thailand have demonstrated a strong link between these environmental factors and the incidence of falls among older adults, as assessed using the Thai-Home Fall Hazard Assessment Tool (Thai-HFHAT) [10]. Indoor environmental factors, particularly uneven house floors, were key contributors to falls among older adults compared to homes with clean and tidy floors in Thailand’s southern border provinces [11]. However, current evidence remains geographically limited and may not reflect the unique risks faced by elderly individuals living in rural coastal areas [12]. 

Coastal regions in southern Thailand, such as Nakhon Si Thammarat, Phatthalung, Songkhla, and Surat Thani, present distinct environmental challenges that elevate fall risk among older adults. Prolonged rainy seasons and persistently high humidity create slippery surfaces, particularly on wooden floors and outdoor walkways, where algae and moss accumulate rapidly. Many traditional homes are wooden stilt houses with open-air living spaces, steep stairs, and outdoor or distant bathing areas, all of which increase exposure to wet and uneven surfaces. Salt-laden coastal air further accelerates corrosion of metal components and deterioration of wooden structures, weakening handrails and flooring more quickly than in inland settings [13]. These conditions contrast with inland rural environments, where falls are more commonly associated with poor lighting, poor pedestrian surfaces, unsafe pathways and general age-related housing deterioration rather than moisture, salinity, or structural corrosion [10, 14]. As a result, older adults in coastal communities face a unique combination of climatic and architectural hazards that collectively heighten fall risk. Despite these distinctive conditions, little is known about how they influence fall risk among older adults. In Ecuador, Orces reported that 11.4% of older adults experienced fall-related injuries, with higher prevalence in urbanized coastal provinces [15]. However, the study did not examine environmental features specific to coastal areas. Similarly, Tsai et al. found a 13.8% fall prevalence in an agricultural and fishing community in southern Taiwan and identified fear of falling and low physical activity as key predictors [16]. Yet, the influence of home environmental hazards unique to coastal living was not addressed.

Collectively, these findings reveal a critical gap in the literature. Therefore, this study aims to investigate the association between home environmental factors and the incidence of falls among older adults living in a rural coastal community in Thailand. Specifically, the study will investigate the housing characteristics and environmental hazards in Surat Thani Province, focusing on the Talat Chaiya Sub-district, which represents coastal southern Thailand due to its high proportion of older adults, mixed local and in-migrant population, and demographic patterns typical of rural coastal communities [2]. The findings aim to provide actionable evidence for region-specific fall prevention programs and support the development of scalable models for use in other coastal areas across the country.

## Methods

### Study design, setting, and sample

A cross-sectional study was carried out with community-dwelling older adults aged 60 years and above living in Talat Chaiya Sub-District, Chaiya District, Surat Thani Province, Thailand. Located in the southern region of Thailand, this province is classified as a coastal rural area. Maps of Talat Chaiya Sub-District, Chaiya District, Surat Thani Province can be accessed through the public website: https://en.wikipedia.org/wiki/Surat_Thani.

Participants were recruited from the registry of the Municipal Elderly School of Chaiya, which comprises elderly individuals officially recognized as residents of the sub-district. The inclusion criteria were: (1) Thai nationals aged 60 years or above who were currently lived in their own homes within the study area; (2) Willingness to provide informed consent and voluntarily participate in the study; and (3) Ability to communicate independently with the researcher—each question was read aloud to participants, who then responded according to their own understanding. Individuals with severe complications from critical illnesses were excluded from the study.

A total of 70 registered members of the Talat Chaiya Municipality School for Older Persons were listed in the municipal registry. The required sample size was determined using Taro Yamane’s (1973) [17] formula for community-based descriptive studies, where the total population size is known and the variables of interest are measured as proportions (e.g., yes/no, ever/never, agree/disagree). The calculation was performed using a 95% confidence level and a permissible error margin of 0.05. The formular used for estimating the sample size as follows:

$$\:n=\frac{N}{1+N{\left(e\right)}^{2}}$$

Where n = sample size at a 95% confidence level.

N = total population (70 older adults).

e = acceptable sampling error (0.05)

$$\:n=\frac{70}{1+70{\left(0.05\right)}^{2}}=59.57$$

Accordingly, the final sample size was rounded to 60 participants. Simple random sampling was applied to select participants from the registry of older adults.

### Ethical approval

The study was conducted in accordance with the guidelines of the Declaration of Helsinki and was approved by the Ethics Committee of Surat Thani Rajabhat University (Approval number: SRU-EC 2024/048). All participants were fully informed about the study’s procedures and objectives and provided written informed consent before taking part.

## Assessments

### Demographic and health conditions

Participants underwent face-to-face, individual interviews administered by four trained research personnel using standardized questionnaires to collect sociodemographic data (age, sex, education level, employment status, and living arrangement) and clinical information, including medical conditions and medication use. All interviewers completed uniform training, and intrarater reliability was confirmed prior to fieldwork to ensure consistency in questionnaire administration.

### History of falls

Participants were asked about the history of fall during the previous six months. A fall was defined as “an unexpected event in which the person comes to rest on the ground, floor, or lower level” [18]. Individuals who experienced at least one such incident during that period were classified as fallers.

### Home environmental assessment

To assess the potential environmental risk of falls, field visits were conducted for on-site evaluations of each older adult’s actual home environment. The assessment employed a structured questionnaire that was adapted from the Clean and Healthy Home Assessment Form [19], the Thai-Home Fall Hazard Assessment Tool (Thai-HFHAT) [20], and recommendations identified in the literature review from the Project to Study Minimum Standards for Housing and Environment for the Elderly [21]. The questionnaires were validated through a rigorous process involving content validity assessment by three subject matter experts and subsequent reliability testing, both yielding satisfactory results.

The questionnaire included closed-ended questions and was structured into four sections: (1) living environment (12 items), (2) bedroom area (10 items), (3) bathroom area (9 items), and (4) kitchen area (6 items). Each item was positively phrased to reflect recommended characteristics of a safe and supportive home environment. Responses for each questionnaire item were recorded using a binary scoring system. A score of 0 was assigned when the assessed area contained five or fewer safety spots, indicating insufficient environmental safety, whereas a score of 1 was given when the area contained more than five safety spots, reflecting a higher level of environmental protection [19–21]. The cutoff of five safety spots was informed by expert consensus developed through group discussions using a Delphi-informed approach, to ensure contextual appropriateness for the local coastal environment [22, 23]. In terms of fall risk interpretation, a score of 0 for any given item was classified as representing a high risk of falling, as it denoted fewer safety spots and, therefore, insufficient environmental protection. In contrast, a score of 1 was classified as representing a low risk of falling, as it reflected the presence of a higher number of safety spots, thereby providing greater environmental safeguards against falls.

### Quality of tools

The interview-based questionnaire was validated through expert review by two university lecturers specializing in nursing and public health, and an environmental health specialist. Following the expert assessment, the content validity was confirmed, with an Item-Objective Congruence (IOC) index ranging from 0.67 to 1.00 and an average IOC value of 0.98, indicating acceptable agreement among experts [24]. Reliability testing was then conducted with 30 age-matched participants using standardized and validated questionnaires. The Cronbach’s alpha coefficient was 0.80, demonstrating satisfactory internal consistency. Inter-rater reliability was also evaluated during the home visits. Using the intra-class correlation coefficient (ICC), the questionnaire demonstrated good inter-rater reliability, with ICC values exceeding 0.80.

### Statistical analyses

Descriptive statistics were used to summarize sociodemographic/individual characteristics information. The Chi-square test was applied to investigate the association between these characteristics, home environmental risk factors, and fall in the older adults. Fisher’s exact test was used when more than 20% of the expected cell counts were less than five [25]. Relationships between variables were analyzed using the Chi-square test, with statistical significance set at a *p*-value of ≤ 0.05. All analyses were conducted using IBM SPSS Statistics version 29.0 (IBM Site Number: 4225416, IBM Corp., Armonk, NY, USA).

## Results

A total of sixty older adults who completed both the general questionnaire and the fall history questionnaire met the inclusion criteria and were included in the study. Participants were then divided into two groups according to whether they had experienced a fall in the past six months: 10 out of 60 participants (17%) reported experiencing at least one fall and were categorized as fallers, while the remaining 50 participants (83%) reported no falls and were classified as non-fallers.

Among the participants, 85% were female, and the median age was 66.5 years (IQR = 11). Overall, 35% were unemployed and 25% lived alone. Additionally, 70% had one or more underlying conditions—including hypertension, diabetes, or dyslipidemia—and 75% reported taking at least one medication. As shown in Table 1, unemployed participants were more likely than employed participants to have experienced one or more falls in the past six months.

**Table 1:** Baseline characteristics of participants were categorized based on their fall history into non-fallers (no falls) and fallers (one or more falls) (*n *= 60)

Characteristic, n (%) or mean ± SD	Category	Fallers (≥ 1) *n* = 10	Non-fallers (0) *n* = 50	*P*-value^*^
Demographics				
Sex	Male	2 (3.3)	7 (11.7)	0.637^†^
	Female	8 (13.3)	43 (71.7)	
Age	60–69 years	5 (8.3)	32 (53.3)	0.485^†^
	70 years and over	5 (8.3)	18 (30)	
Median = 66.5, IQR = 11, Max = 93, Min = 60				
Career status	Unemployed	8 (13.3)	13 (21.7)	**0.002**^†^
	Employed	2 (3.3)	37 (61.7)	
Education level	Uneducated	0 (0)	4 (6.7)	1.000^†^
	Educated	10 (16.7)	46 (76.7)	
Living type	Living alone	3 (5)	12 (20)	0.700^†^
	Living with family	7 (11.7)	38 (63.3)	
One or more underlying condition	Yes	8 (13.3)	34 (56.7)	0.708^†^
	No	2 (3.3)	16 (26.7)	
One or more medication used	Yes	9 (15)	36 (60)	0.426^†^
	No	1 (1.7)	14 (23.3)	

*n* = number of participants, *IQR =* Interquartile Range. Underlying condition refers to conditions self-reported as medically diagnosed. Educated refers to individuals with formal education ranging from primary school to a Bachelor's degree

^*^Chi-square tests were performed for categorical variables, with significant results (*p* < 0.05) indicated in bold

^†^Fisher’s exact test was used when more than 20% of the expected cell counts were less than 5 ^25^

Table 2 presents the finding of the Chi-Square test examining four components of home environmental risk factors: living environment, bedroom area, bathroom area, and kitchen area. A significant relationship was observed between the presence of mosquito nets and the risk of falls. Among participants with high environmental fall risk, non-fallers were significantly more likely to report using a mosquito net in the bedroom compared to fallers (*X*^*2*^ (1, *N* = 60) = 5.676, *p* = 0.031) (Table 2).

**Table 2:** Association between home environmental hazards and fall occurrences among older adults (*n* = 60), analyzed using Fisher’s exact test

**Home environmental risk factors**	**Risk level**	**Fallers** **n (%)**	**Non - fallers** **n (%)**	**Chi-Square Test**^†^	*p*-value^*^
Living environment					
1. Structurally sound house	High	7 (11.7)	40 (66.7)	0.491	0.675
	Low	3 (5)	10 (16.7)		
2. Clean and organized interior	High	9 (15.0)	43 (71.7)	0.115	1.000
	Low	1 (1.7)	7 (11.7)		
3. Well-ventilated and naturally lit	High	8 (13.3)	42 (70)	0.096	0.668
	Low	2 (3.3)	8 (13.3)		
4. Smooth walkways and flooring	High	7 (11.7)	47 (78.3)	5.333	0.052
	Low	3 (5)	3 (5)		
5. Accessible door thresholds	High	9 (15)	44 (73.3)	0.032	1.000
	Low	1 (1.7)	6 (10)		
6. Handrails on walkways	High	9 (15)	46 (76.7)	0.044	1.000
	Low	1 (1.7)	4 (6.7)		
7. Well-lit entrance	High	6 (10)	43 (71.7)	3.763	0.074
	Low	4 (6.7)	7 (11.7)		
8. Automatic circuit breaker	High	7 (11.7)	40 (66.7)	0.491	0.675
	Low	3 (5)	10 (16.7)		
9. Safe furniture design	High	8 (13.6)	45 (76.3)	1.274	0.266
	Low	2 (3.4)	4 (6.8)		
10. Visible indoor color scheme	High	6 (10)	44 (73.3)	4.704	0.052
	Low	4 (6.7)	6 (10)		
11. Handrails on staircase	High	10 (16.7)	46 (76.7)	0.857	1.000
	Low	0 (0)	4 (6.7)		
12. Clear furniture arrangement	High	8 (13.3)	45 (75.0)	0.809	0.330
	Low	2 (3.3)	5 (8.3)		
Bedroom area					
1. Proper bed height and placement	High	7 (11.7)	39 (65)	0.298	0.685
	Low	3 (5)	11 (18.3)		
2. The presence of mosquito wire screens or mosquito nets	High	5 (8.3)	42 (70)	5.676	**0.031**
	Low	5 (8.3)	8 (13.3)		
3. Clean bed and linens	High	9 (15)	43 (71.7)	0.115	1.000
	Low	1 (1.7)	7 (11.7)		
4. Stable furniture without sharp edges	High	7 (11.7)	42 (70)	1.091	0.371
	Low	3 (5)	8 (13.3)		
5. Operable windows for ventilation	High	6 (10)	43 (71.7)	3.763	0.074
	Low	4 (6.7)	7 (11.7)		
6. Adequate bedroom lighting	High	6 (10)	42 (70)	3.000	0.101
	Low	4 (6.7)	8 (13.3)		
7. Level floors without thresholds	High	8 (13.3)	43 (71.7)	0.235	0.637
	Low	2 (3.3)	7 (11.7)		
8. Stable table and chairs	High	7 (11.7)	44 (73.3)	2.118	0.163
	Low	3 (5)	6 (10)		
9. Bed handrails	High	6 (10)	34 (56.7)	0.240	0.718
	Low	4 (6.7)	16 (26.7)		
10. Proper shelf height	High	10 (16.7)	48 (80)	0.414	1.000
	Low	0 (0)	2 (3.3)		
Bathroom area					
1. Non-slip bathroom floor and mat	High	9 (15)	47 (78.3)	0.214	0.528
	Low	1 (1.7)	3 (5)		
2. Sturdy seated toilet	High	9 (15)	44 (73.3)	0.032	1.000
	Low	1 (1.7)	6 (10)		
3. Bathroom grab bars	High	5 (8.3)	38 (63.4)	2.774	0.128
	Low	5 (8.3)	12 (20)		
4. Bathroom ventilation	High	7 (11.7)	42 (70)	1.091	0.371
	Low	3 (5)	8 (13.3)		
5. Adequate bathroom lighting	High	9 (15)	43 (71.6)	0.115	1.000
	Low	1 (1.7)	7 (11.7)		
6. Accessible bathroom storage	High	8 (13.3)	38 (63.3)	0.075	1.000
	Low	2 (3.3)	12 (20)		
7. Sliding or outward-opening door	High	7 (11.7)	43 (71.7)	1.536	0.347
	Low	3 (5)	7 (11.7)		
8. Separated wet and dry bathroom areas	High	10 (16.7)	45 (75)	1.091	0.578
	Low	0 (0)	5 (8.3)		
9. Bathroom near bedroom	High	8 (13.3)	43 (71.7)	0.235	0.637
	Low	2 (3.3)	7 (11.7)		
Kitchen area					
1. Smooth non-slip kitchen floor	High	9 (15)	45 (75)	0.000	1.000
	Low	1 (1.7)	5 (8.3)		
2. Adequate kitchen lighting	High	8 (13.3)	41 (68.3)	0.022	1.000
	Low	2 (3.3)	9 (15)		
3. Safe stove type	High	8 (13.3)	33 (55)	0.775	0.480
	Low	2 (3.3)	17 (28.3)		
4. Organized kitchen storage	High	7 (11.7)	41 (68.3)	0.750	0.403
	Low	3 (5)	9 (15)		
5. Elevated kitchen workbench	High	7 (11.7)	41 (68.3)	0.750	0.403
	Low	3 (5)	9 (15)		
6. Clean dry kitchen floor	High	10 (16.7)	43 (71.7)	1.585	0.589
	Low	0 (0)	7 (11.7)		

^†^Fisher’s exact test

*p-value statistically significant at ≤ 0.05

### Discussion

Among older adults residing in rural coastal areas, 17% reported having at least one fall within the previous six months. Notably, individuals who were unemployed exhibited a higher prevalence of falls during this period compared to those who were employed. Additionally, the presence of a mosquito net in the bedroom was significantly associated with a reduced risk of falling.

The fall prevalence reported in this study is slightly higher than that observed among older adults living in the non-coastal regions of southern Thailand, where a recent study documented a 6-month fall prevalence of 12.1% [26]. By contrast, the prevalence observed in our coastal community sample exceeded this figure, suggesting that falls may be more common in coastal than in non-coastal regions. This interpretation is supported by international evidence. For example, a cohort study in Hunei, northern Kaohsiung, Taiwan, reported a 12-month fall prevalence of 13.8% among older adults in agricultural and fishing communities [16]. Likewise, in Ecuador, a household survey found a 12-month prevalence of fall-related injury of 11.4% among older adults in coastal regions, compared with lower estimates in highland (Andean) areas [15]. Such variations highlight the influence of local environmental factors, such as housing characteristics, outdoor terrain, and access to age-friendly infrastructure on fall prevalence across regions.

In this study, being unemployed was associated with a higher risk of falls among older adults residing in coastal rural areas. This finding aligns with Bhoomika et al. (2022), who reported that unemployment was linked to a higher risk of falls among older adults in rural India [27], and with Juntasopeepun and Bliss (2025), who demonstrated similar patterns among community-dwelling older adults in Thailand [28]. Several mechanisms may account for this association. Unemployed older adults often spend more time at home, increasing their exposure to domestic hazards and activities that may precipitate falls. They may also have fewer opportunities to participate in structured exercise or community programs that promote balance, strength, and mobility confidence [29]. From a broader theoretical perspective, unemployment in later life has been repeatedly associated with elevated frailty risk, reduced activity participation, and diminished social engagement—factors that accelerate physiological decline and heighten vulnerability to falls [30]. Evidence from Japan, the UK, and Taiwan demonstrates that continued engagement in work or socially productive activities helps preserve functional capacity, supports physical and psychosocial well-being, and mitigates frailty progression, whereas unemployment predicts lower activity levels and greater frailty development over time [30–32]. These patterns indicate that frailty-related pathways may underpin the unemployment–fall relationship observed in our study. In addition, unemployment often coincides with lower socioeconomic status, which can further limit access to healthcare, assistive devices, and home modifications that support fall prevention [33, 34]. Notably, a recent study found that older adults engaged in agricultural work in rural coastal areas experienced a lower risk of falls, underscoring the potential protective influence of sustained employment or productive activity [35]. Together, these findings highlight the importance of promoting opportunities for continued engagement in work or meaningful social and economic activities to support functional health, reduce frailty, and thereby lower fall risk among older adults.

Our finding that the presence of mosquito screens or nets in the bedroom was associated with a lower risk of falls among older adults in rural coastal areas should be interpreted cautiously. While the mechanism remains unclear, several hypotheses may explain this association. One possibility is that the installation of mosquito screens contributes to a tidier and less cluttered home environment, as windowsills and doorways may be kept clear, thereby potentially reducing trip hazards—an exposure that has been linked to fall risk in previous studies [7, 10]. Another hypothesis is that mosquito screens may allow greater use of natural light during the day, which could improve visibility indoors; inadequate bedroom lighting has been associated with increased fall risk, whereas sufficient lighting is known to enhance gait performance and reduce step variability [36, 37]. Additionally, mosquito screens or nets may facilitate better ventilation and airflow—particularly relevant in coastal homes that often lack air conditioning—allowing residents to keep windows open without concern for insects [38]. Improved airflow may contribute to greater comfort and potentially better sleep quality, which has been associated with reduced fatigue and improved balance [39]. These proposed explanations remain hypothetical, and further research is required to elucidate the underlying mechanisms driving this observed association.

A key strength of this research lies in its thorough assessment of home environmental risk factors contributing to falls, with particular attention to older adults living in rural coastal areas. However, a few limitations should be noted. First, the use of fall questionnaire-based interviews over the past six months may introduce recall and social desirability bias, as falls are often under-reported due to memory difficulties, stigma, and differences in how they are defined [40, 41]. We sought to minimise this risk by using a clear, standardised definition of falls and have no reason to believe it affected low- and high-risk participants differently. Second, the relatively small sample size and recruitment from a single province limit the generalizability of the findings to other coastal or inland regions of Thailand. Third, the cross-sectional design precludes causal inference; while the study identifies associations between environmental factors, employment status, and fall history, temporal relationships cannot be established. Future research should utilise larger, more diverse samples and longitudinal or prospective fall surveillance to confirm these associations and better clarify the causal pathways involved.

## Recommendations and conclusions

This study identifies notable home-environment and individual-level factors associated with falls among older adults living in rural coastal communities. The observed protective association of having a mosquito net in the bedroom suggests that practical and low-cost modifications to the home environment may contribute meaningfully to fall prevention efforts. In addition, sociodemographic characteristics—particularly employment status—were relevant, with unemployed individuals showing a higher six-month fall prevalence than those who were employed. These findings emphasize the need to consider both environmental and personal determinants when developing fall-prevention strategies for older adults in under-resourced settings.

In light of these results, it may be beneficial to integrate routine home-environment risk screening into existing community-based ageing initiatives, such as Thailand’s Municipal Elderly School or the Village Health Volunteer program, to strengthen early identification and timely risk management. Embedding such assessments within established public health structures could enhance feasibility, broaden reach, and support sustainable fall-prevention practices at the community level.

Finally, although this study contributes valuable insight into factors associated with falls in this population, the cross-sectional nature of the data limits the ability to infer causality. Future research would benefit from longitudinal or interventional study designs to verify the observed associations, determine temporal relationships, and evaluate the effectiveness of specific environmental or behavioral interventions aimed at reducing fall risk among older adults in rural areas.

## Data Availability

The data supporting the findings of this study are available from the corresponding author upon reasonable request.
